# Paths, negative “probabilities”, and the Leggett-Garg inequalities

**DOI:** 10.1038/s41598-019-43528-5

**Published:** 2019-05-08

**Authors:** D. Sokolovski, S. A. Gurvitz

**Affiliations:** 1 Departmento de Química-Física, Universidad del País Vasco, UPV/EHU, Leioa, Spain; 2 IKERBASQUE, Basque Foundation for Science, 48013 Bilbao, Spain; 3 Department of Particle Physics and Astrophysics, Weizmann Institute of Science, Rehovot, 76100 Israel

**Keywords:** Quantum physics, Information theory and computation

## Abstract

We present a path analysis of the condition under which the outcomes of previous observation affect the results of the measurements yet to be made. It is shown that this effect, also known as “signalling in time”, occurs whenever the earlier measurements are set to destroy interference between two or more virtual paths. We also demonstrate that Feynman’s negative “probabilities” provide for a more reliable witness of “signalling in time”, than the Leggett-Garg inequalities, while both methods are frequently subject to failure.

## Introduction

Recently, the authors of^1^ have shown that superconducting flux qubits possess, despite their macroscopic nature, such quantum properties, as the ability to exist in a superposition of distinct states. After reviewing an approach based on the so-called Leggett-Garg inequalities (LGI), which may or may not be satisfied by certain quantum mechanical averages^2^, they chose to employ a simpler experimental protocol. The method used in^1^ was similar to the one proposed by Koffler an Bruckner^3^, who suggested that the relevant evidence can be obtained more efficiently by analysing corresponding probability distributions, and coined a term “signalling in time”.

Both the LGI, and the notion of “signalling”, are closely related to a different problem, the so-called Bell test^4^, in which Alice an Bob are given two spins in the zero total spin state. Alice’s measurement along a chosen axis immediately aligns Bob’s spin in the opposite direction, and the study of inequalities, formally similar to the LGI, allowed Bell to show that the phenomenon cannot be explained by the existence of certain classical-like hidden variables. There is, however, no “signalling” in the Bell’s experiment, in the sense that Bob is unable to recognise the choice of the axis made by Alice. Reduced density matrix of the Bob’s spin is not affected by Alice’s decision, and the no-cloning theorem^5^, constraints his ability to reconstruct the spin’s state.

Feynman’s approach to Bell’s problem has been more direct. In the^6^ he demonstrated that, in order to reproduce quantum results for an entangled Bell’s state with hidden variables, some the probabilities would inevitably turn negative. In a recent essay on the relationship between Feynman and Bell^7^, Whitaker notes that “what Feynman describes is indeed Bell’s Theorem”. A similar, yet somewhat different approach to the “signalling in time” problem was recently proposed in^8^, where negative values taken by quasi probabilities, defined in terms of quantum projection operators, were related with violations of the LGI.

Several authors^3,8,9^, emphasise the difference between the Bell’s case, and the problem, to which the LGI is usually applied. Indeed, here one makes several consecutive measurements on the same quantum system, and asks whether the outcomes of previous observations can influence the results of the measurements yet to be made. Interaction with a measurement device at some *t*_1_ can scatter the system, at ${t}_{2} > {t}_{1}$, into a state it would not have visited otherwise, or would visit it with a different frequency. Should this happen, “signalling in time” is said to have occurred (We note that “signalling in time” may be a rather fancy description of what happens. A tennis player, returning the ball the his/her partner may be said to “have sent a signal forward in time”. However, “hitting the ball back” would usually do). No “signalling” means that a measurement does not change the outcome statistics of later measurements^3^.

The literature on the Leggett-Garg inequalities is extensive, and we refer the reader to a recent review^10^ for relevant references, covering different aspects of the problem. The scope of this paper is much narrower. First, we analyse “signalling in time” in terms of the virtual (Feynman) paths, and illustrate the analysis on the simple example of a qubit undergoing Rabi oscillations. Having done so, we compare the Feynman’s direct “negative probability” test, and the violation of the LGI, as possible indicators of the “signalling” phenomenon.

## Path Analysis of “Signalling in Time”

Consider a sequence of accurate measurements of quantities {${\hat{Q}}_{1}$, ${\hat{Q}}_{2}$, …, ${\hat{Q}}_{K}$} which could, in principle, be made on a quantum system in a Hilbert space of a dimension *N* at different times, {*t*_1_, *t*_2_, …, *t*_*K*_}. Usually, the problem is treated as follows (see, for example^11^). The quantities of interest are represented by operators with spectral decompositions ${\hat{Q}}_{k}={\sum }_{{Q}_{k}}\,{Q}_{k}\hat{\pi }({Q}_{k})$, ${\sum }_{{Q}_{k}}\,\hat{\pi }({Q}_{k})=1$. In what follows, we will assume all eigenvalues *Q*_*k*_ to be distinct, ${\hat{\pi }}_{{Q}_{k}}=|{Q}_{k}\rangle \,\langle {Q}_{k}|$, and refer the reader to^12^ for a more general analysis. If the system is in the state $|\psi \rangle$  at t = 0,  prior to the first measurement, the probability to obtain results ${Q}_{1},{Q}_{2},\ldots ,{Q}_{K}$ is written as an average^11^,

1 $$P({Q}_{1},{Q}_{2},\ldots ,{Q}_{K})=\langle \psi |{\hat{\pi }}_{{Q}_{1}}^{\dagger }({t}_{1}){\pi }_{{Q}_{2}}^{\dagger }({t}_{2})\ldots {\pi }_{{Q}_{K}}^{\dagger }({t}_{K}){\pi }_{{Q}_{K}}({t}_{K})\ldots {\pi }_{{Q}_{2}}({t}_{2}){\pi }_{{Q}_{1}}({t}_{1})|\psi \rangle $$

where the information about temporal developments is contained in the projectors, ${\hat{\pi }}_{{Q}_{k}}({t}_{k})={\hat{U}}^{\dagger }({t}_{k}){\hat{\pi }}_{{Q}_{k}}\hat{U}({t}_{k})$, $\hat{U}(t)\equiv \exp (\,-\,i\hat{H}t)$, given here in the Heisenberg representation.

For us it will be more convenient to see a measurements outcome as a result of interference between various scenarios, which unfold as the time progresses. In the spirit of Feynman’s path integral^6^, we will call a path a sequence of possible outcomes (numbers), {*Q*_1_, *Q*_2_, …, *Q*_*K*_}, where *Q*_*i*_, $1\le i\le K$, is one of the eigenvalues of the operator ${\hat{Q}}_{i}$. (The simplest path would connect just two outcomes, e.g., *Q*_2_ and *Q*_1_). For every path quantum mechanics provides a complex valued *probability amplitude*, $A({Q}_{1},{Q}_{2},\ldots ,{Q}_{K})$, given in our case by

2 $$A({Q}_{1},{Q}_{2},\ldots ,{Q}_{K})=\langle {Q}_{K}|\hat{U}({t}_{K}-{t}_{K-1})|{Q}_{K-1}\rangle \,\langle {Q}_{K-1}|\ldots |{Q}_{2}\rangle \,\langle {Q}_{2}|\hat{U}({t}_{2}-{t}_{1})\,|{Q}_{1}\rangle \langle {Q}_{1}|\hat{U}({t}_{1})|\psi \rangle .$$

If all of the measurements are actually made, quantum mechanics provides also the probabilities^13^, and Eq. (1) can be rewritten as $P({Q}_{1},{Q}_{2},\ldots ,{Q}_{K})=|A({Q}_{1},{Q}_{2},\ldots ,{Q}_{K}){|}^{2}$. The probabilities are related to the frequencies, with which a given sequence is be observed, as the system is seen to “travel” the corresponding path. While paths endowed only with probability amplitudes are usually called *virtual*, we will refer to the paths, to which both the amplitude and the probability can be assigned, as *real*^14^. We will call the set of all relevant real paths, together with the corresponding probabilities, a *statistical ensemble*. The probabilities for real paths can be obtained by adding the probability amplitudes of the virtual paths, and taking the absolute square, as appropriate^13^. Importantly, choosing to make different measurements from the set {${\hat{Q}}_{1}$, ${\hat{Q}}_{2}$, …, ${\hat{Q}}_{K}$} may lead to essentially different statistical ensembles^12,14^. For example, if a measurement at $t={t}_{k}$ $1\le k < K$ is not made, the amplitude for the remaining $K-1$ outcomes is a coherent sum of the amplitudes (2),

3 $$A({Q}_{1},\ldots {Q}_{k-1},{Q}_{k+1}\ldots ,{Q}_{K})=\sum _{{Q}_{k}}\,A({Q}_{1},\ldots {Q}_{k-1},{Q}_{k},{Q}_{k+1}\ldots {Q}_{K}).$$

A decision not to make the last measurement at $t={T}_{K}$ may not affect the measurements already made. In this case, one recovers the amplitude in Eq. (2), but for a shorter path {*Q*_1_, *Q*_2_, …, *Q*_*K*−1_}, and the probability

4 $$P({Q}_{1},{Q}_{2},\ldots ,{Q}_{K-1})=|A({Q}_{1},{Q}_{2},\ldots ,{Q}_{K-1}){|}^{2}.$$

Now the problem of “signalling in time” can be seen as follows. Two measurements at ${t}_{1} < {t}_{3}$ produce an ensemble with *N*^2^ real paths. Adding a third measurement at ${t}_{1} < {t}_{2} < {t}_{3}$ yields another ensemble, with *N*^3^ real paths. This ensemble is different, i.e. incompatible, with the first one, in the sense that ignoring the outcomes at *t*_2_ (non-selective measurement), and adding the corresponding probabilities, does not recover the ensemble, obtained with the measurements made at *t*_1_ and *t*_3_ only. Incompatibility of different ensembles comes as a natural consequence of the fact that one cannot always avoid perturbing an accurately measured quantum system.

## Virtual and Real Paths for a Qubit

**Figure 1:**
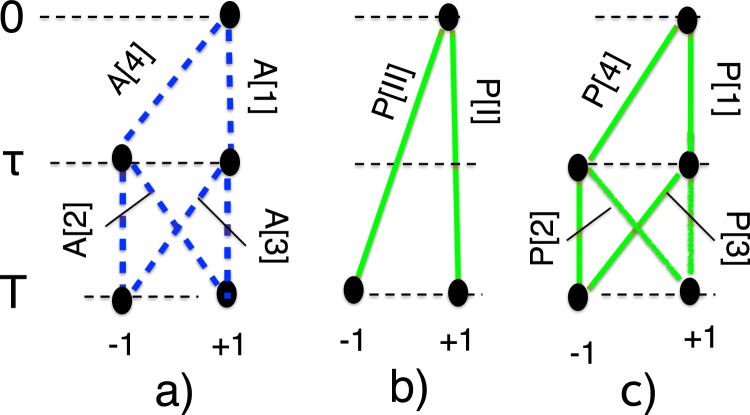
(**a**) Four virtual paths in Eq. (5), for the chosen sets of measurements; (**b**) real paths in Eq. (8); (**c**) real paths in Eq. (9).

As an example, consider a two-level system, such as spin-1/2 (a qubit), and three consecutive measurements, made at $t=0$, $t=\tau$, and $t=T$, of the same quantity $\hat{Q}=|1\rangle \,\langle 1|-|-\,1\rangle \,\langle -\,1|$, whose possible values are ±1. For simplicity, we will assume that at $t=0$ the system is prepared in an eigenstate of $\hat{Q}$, $|\psi \rangle =|1\rangle$, so that the result of the first measurement is always ${Q}_{1}=+\,1$. Now there are four virtual paths shown in Fig. 1a, which we will label 1, 2, 3 and 4 to avoid lengthy notations,

5 $$\begin{array}{ll}\{1\}\equiv \{1,1,1\}, & \{2\}\equiv \{1,-\,1,1\},\\ \{3\}\equiv \{\,-\,1,1,1\}, & \{4\}\equiv \{\,-\,1,-\,1,1\}.\end{array}$$

For a system, performing Rabi oscillations of a unit frequency between the states $|1\rangle$ and $|\,-\,1\rangle$, the paths are endowed with the probability amplitudes

6 $$\begin{array}{ccc}A[1] & \equiv  & \langle 1|\hat{U}(T-\tau )|1\rangle \,\langle 1|\hat{U}(\tau )|1\rangle =\,\cos (T-\tau )\,\cos \,\tau ,\\ A[2] & \equiv  & \langle 1|\hat{U}(T-\tau )|-\,1\rangle \,\langle \,-\,1|\hat{U}(\tau )|1\rangle =-\,\sin (T-\tau )\,\sin \,\tau ,\\ A[3] & \equiv  & \langle \,-\,1|\hat{U}(T-\tau )|1\rangle \,\langle 1|\hat{U}(\tau )|1\rangle =-\,i\,\sin (T-\tau )\,\cos \,\tau ,\\ A[4] & \equiv  & \langle \,-\,1|\hat{U}(T-\tau )|-\,1\rangle \,\langle \,-\,1|\hat{U}(\tau )|1\rangle =-\,i\,\cos (T-\tau )\,\sin \,\tau ,\end{array}$$

where

7 $$\hat{U}(t)=\,\cos \,(t)\hat{I}-i\,\sin \,(t){\hat{\sigma }}_{x},$$

and $\hat{I}$ and ${\hat{\sigma }}_{x}$ are the unity, and the Pauli *x*-matrix, respectively.

We will consider two sets of measurements,

(i) made at ${t}_{1}=0$ and ${t}_{3}=T$, yielding ${Q}_{1}=1$ and some *Q*_3_, and
(ii) made at ${t}_{1}=0$, ${t}_{2}=\tau$ and ${t}_{3}=T$, yielding ${Q}_{1}=1$, *Q*_2_ and *Q*_3_. The corresponding statistical ensembles are shown in Fig. 1b,c. In the case (i) there are just two real paths, $\{I\}=\{1,1\}$ and $\{II\}=\{\,-\,1,1\}$, given by the superpositions of the paths of {1} and {2}, and of {3} and {4}, respectively (see Fig. 1b). The corresponding probabilities, therefore, are

8 $$P[I]=|A[1]+A[2]{|}^{2}={\cos }^{2}(T),$$

$$P[II]=|A[3]+A[4]{|}^{2}={\sin }^{2}(T).$$

In the case (ii), all four paths in Eq. (5) become real (see Fig. 1c), and are travelled with the probabilities

9 $$\begin{array}{ll}P[1]={\cos }^{2}(T-\tau )\,{\cos }^{2}(\tau ), & P[2]={\sin }^{2}(T-\tau )\,{\sin }^{2}(\tau ),\\ P[3]={\sin }^{2}(T-\tau )\,{\cos }^{2}(\tau ), & P[4]={\cos }^{2}(T-\tau )\,{\sin }^{2}(\tau ).\end{array}$$

Our aim is to identify the conditions under which the ensembles in Fig. 1 are found to be essentially different. In other words, like other authors before us^2,3,8–10^, we want to know when making a measurement of *Q*_2_ at $t=\tau$ affects the distribution of the values *Q*_3_ at $t=T$. It is sufficient to compare the probabilities to have ${Q}_{3}=1$ in the cases b) and c) shown in Fig. 1,

10 $$\begin{array}{rcl}{Pro}{{b}}_{b}({Q}_{3}=1) & = & P[I]={\cos }^{2}(T)\\ {Pro}{{b}}_{c}({Q}_{3}=1) & = & P[1]+P[2]={\cos }^{2}(T)+\frac{1}{2}\,\sin (2\tau )\,\sin (2(T-\tau ))\end{array}$$

**Figure 2:**
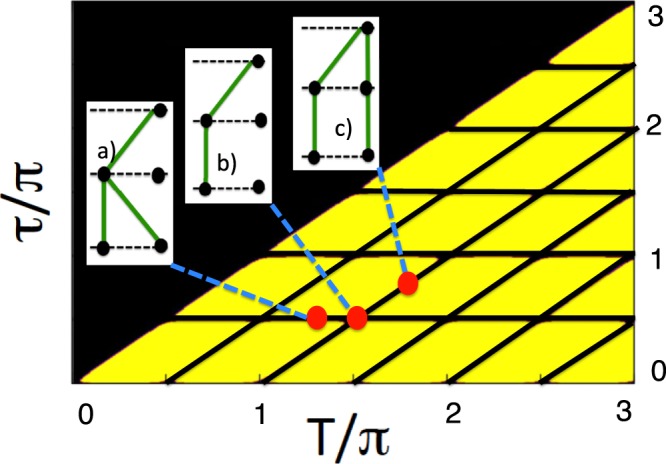
Detection of “signalling in time” based on Eq. (10). In the light coloured areas $\delta P(\tau ,T)\ne 0$, and the ensemble consists of the four real paths shown in Fig. 1c. On the dark coloured horizontal and diagonal lines, the are only two real paths [see insets (**a** and **c**)]. At their intersection, there is only one real path, shown in the inset (**b**).

In Fig. 2, we plot the difference, $\delta P(\tau ,T)\equiv {Pro}{{b}}_{c}({Q}_{3}=1)-{Pro}{{b}}_{b}({Q}_{3}=1)$, brought about by the disturbance produced by the measurement at $t=\tau$. The two probabilities agree, $\delta P(\tau ,T)=0$, only if $\tau$ or $T-\tau$ equals *nπ*/2, $n=0,1,2\ldots$, i.e., where two or three of the amplitudes in Eq. (6) vanish. (An analogy with the Young’s interference experiment may be helpful. Treating the states at $\tau$ and *T* as two “slits”, and two “positions on the screen”, respectively, we note that the first case corresponds to one of the slits blocked. Now the paths leading to final positions can be identified without destroying the pattern on the screen. The case $\sin (2(T-\tau )))=2\,\sin (T-\tau )\,\cos (T-\tau )=0$ corresponds to both slits being open, but just one path [{1} and {4}, if $\sin (T-\tau )=0$, or {2} and {3}, if $\cos (T-\tau )=0$] connecting each slit with each final position. Again, the “which way?” question can be answered, since there is no interference to destroy). Thus, in the light-coloured regions in Fig. 2, behaviour of the system can be described as *“quantum stochastic”*. A measurement, added at $t=\tau$ changes the ensemble in Fig. 1b into the one shown in Fig. 1c, the number of real paths increases to four, and the odds for arriving in the final states at $t=T$, are clearly not what they were before. On the horizontal and diagonal dark lines, the system is *“classical stochastic”*. It has only two non-interfering paths^15^, leading to different final destinations [see insets a) and c) in Fig. 2]. There is no interference to destroy and, as in classical statistics, one can monitor the system’s progress, without disturbing it. Finally, at the intersection of any two lines, we may call the system’s behaviour *“classical deterministic”*. There a single path [see inset b) in Fig. 2], which leads to a unique final state, and is travelled every time the experiment is repeated, regardless of whether the measurement at $t=\tau$ is made, or not. Note that this classification refers to a particular choice of measurements, and choosing a different measured operator, or a different initial state, would result in a picture, different from the one shown in Fig. 2.

In general, we note that there can be no pre-determined values (or average values) of *Q*_3_, independent of what being done at $\tau$. Rather, we must conclude that different sets of measurements may “fabricate” completely different statistical ensembles from the same quantum system^12^.

## No “Pre-Existing” Path Probabilities

Next we expand on the last statement of the previous Section. Let us assume (incorrectly) that there are probabilities of having classical-like pre-determined values of ${Q}_{i}=\pm \,1$ at *t*_*i*_. These may depend on unknown classical parameters $\underline{\lambda }$, [as in^4^ we allow multiples, $\underline{\lambda }=({\lambda }_{1},{\lambda }_{2}\ldots {\lambda }_{M})$]. With $\underline{\lambda }$ distributed according to some $w(\mathop{\lambda }\limits_{\_})\ge 0$, $\int w(\underline{\lambda })d\underline{\lambda }=1$, the probabilities for the sequences of the outcomes {*Q*_3_, *Q*_2_, ${Q}_{1}=1$}, can be written as ($d\underline{\lambda }=d{\lambda }_{1}d{\lambda }_{2}\ldots d{\lambda }_{M}$)

11 $$\begin{array}{rcl}p[1] & = & \int {p}_{3}(1|1,1,\underline{\lambda }){p}_{2}(1|1,\underline{\lambda })w(\underline{\lambda })d\underline{\lambda },\\ p[2] & = & \int {p}_{3}(1|-\,1,1,\underline{\lambda }){p}_{2}(\,-\,1|1,\underline{\lambda })w(\underline{\lambda })d\underline{\lambda },\\ p[3] & = & \int {p}_{3}(\,-\,1|1,1,\underline{\lambda }){p}_{2}(1|1,\underline{\lambda })w(\underline{\lambda })d\underline{\lambda },\\ p[4] & = & \int {p}_{3}(\,-\,1|-\,1,1\underline{\lambda }){p}_{2}(\,-\,1,1|\underline{\lambda })w(\underline{\lambda })d\underline{\lambda }.\end{array}$$

In Eq. (11), ${p}_{2}({Q}_{2}|{Q}_{1}=1,\underline{\lambda })$ stands for the probability to have an outcome *Q*_2_, given a previous outcome *Q*_1_, and ${p}_{3}({Q}_{3}|{Q}_{2},{Q}_{1}=1,\underline{\lambda })$ yields the odds for having a value *Q*_3_, given the previous values of *Q*_2_ and *Q*_1_. (Recall that *Q*_1_ is always 1, since the system is prepared in $|1\rangle$). We expect to have no access to the current actual value(s) of the “hidden variable(s)” $\underline{\lambda }$. It is assumed, however, that each time the system is set to evolve from its initial state, particular path probabilities ${p}_{3}({Q}_{3}|{Q}_{2},{Q}_{1},\underline{\lambda }){p}_{2}({Q}_{2}|{Q}_{1},\underline{\lambda })$ exist, even if no measurements are made.

For our assumption to be correct, we need to demonstrate that the classical probabilities (small *p*’s) in Eq. (11) are the same as the correct quantum results (capital *P*’s) of the previous Section. Firstly, we must have

12 $$p[i]=P[i],\,i=1,2,3,4.$$

Secondly, summing the *p*[*i*]’s over the outcomes at $t=\tau$ we should obtain the probabilities *P*(*I*) and *P*(*II*) in Eq. (8), i.e.,

13 $$\begin{array}{rcl}{Prob}({Q}_{3}=1) & = & P[I]=p[1]+p[2],\\ {Prob}({Q}_{3}=-\,1) & = & P[II]=p[3]+p[4],\end{array}$$

However, Eq. (10) states that, in general, $P[1]+P[2]\ne P(I)$, so that Eqs (12) and (13) cannot always hold.

This, in turn, demonstrates, that the path probabilities cannot “pre-exist” a set of consecutive measurements, just as the result of an individual measurement cannot pre-exist the measurement itself^16^. Different measurements may produce statistical ensembles with distributions as different as the distributions of heads and tails for differently skewed coins. This will happen whenever an additional earlier measurement destroys interference between virtual paths leading to later outcomes.

## The Negative Probability Test

We could look for other proofs of the same point, e.g., by following Feynman’s example, described in^6^. We will not rely on a particular type of quasi-probabilities, as was done, for example, in^8^, but rather assume that the classical-like path probabilities *p*[*i*], similar to those in (11), can somehow be defined. We will then look for the values they must take in order to reproduce correct quantum mechanical results. With the help of Eq. (11) it is easy to express the average values of the products, ${\langle {Q}_{i}{Q}_{j}\rangle }_{cl}$, in terms of the *p*[*i*]’s,

14 $$\begin{array}{rcl}{\langle {Q}_{1}{Q}_{2}\rangle }_{cl} & = & p[1]-p[2]+p[3]-p[4],\\ {\langle {Q}_{1}{Q}_{3}\rangle }_{cl} & = & p[1]+p[2]-p[3]-p[4],\\ {\langle {Q}_{2}{Q}_{3}\rangle }_{cl} & = & p[1]-p[2]-p[3]+p[4].\end{array}$$

Using the path probabilities in Eqs (9) and (10) yields the correct quantum value for the same quantities

15 $$\begin{array}{rcl}\langle {Q}_{1}{Q}_{2}\rangle  & \equiv  & P^{\prime} [I]-P^{\prime} [II]=\,\cos (2\tau )\equiv \alpha ,\\ \langle {Q}_{1}{Q}_{3}\rangle  & \equiv  & P[I]-P[II]=\,\cos (2T)\equiv \beta ,\\ \langle {Q}_{2}{Q}_{3}\rangle  & \equiv  & P[1]-P[2]-P[3]+P[4]=\,\cos (2(T-\tau ))\equiv \gamma ,\end{array}$$

If the probabilities (11) exist, results (14) and (15) will agree. Equating ${\langle {Q}_{i}{Q}_{j}\rangle }_{cl}=\langle {Q}_{i}{Q}_{j}\rangle$, and adding a condition

16 $$p[1]+p[2]+p[3]+p[4]=1,$$

yields four linear equations, which the probabilities *p*[*i*] must satisfy. Their solutions are

17 $$\begin{array}{rcl}p[1] & = & (\alpha +\beta +\gamma +1)/4,\\ p[2] & = & (\,-\,\alpha +\beta -\gamma +1)/4,\\ p[3] & = & (\alpha -\beta -\gamma +1)/4,\\ p[4] & = & (\,-\,\alpha -\beta +\gamma +1)/4.\end{array}$$

**Figure 3:**
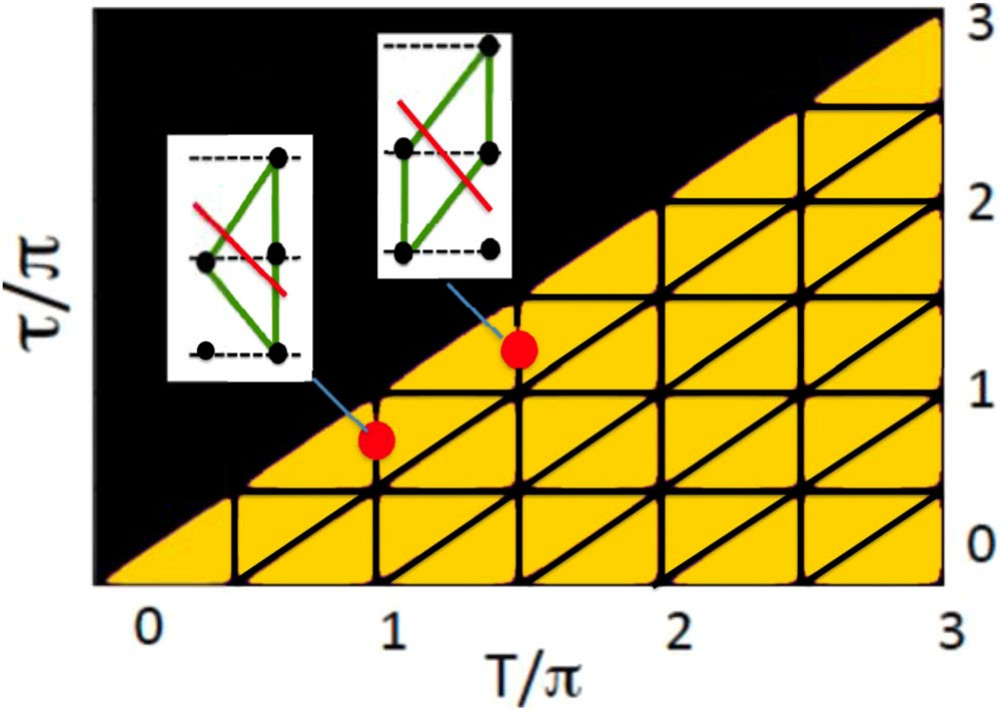
Detection of “signalling in time” by the negative probability test. In the light coloured areas $\delta p(\tau ,T)\ne 0$, while on the dark coloured lines one can find classical path probabilities, consistent with the quantum averages (15). The ensembles, found by the test on the vertical lines (see the insets) are, however, incorrect. The true ensembles for these values of $\tau$ and *T* are those shown in Fig. 1c.

Our assumption will be proven wrong if, for example, some of the *p*[*i*]’s turned out to be negative. Thus, we evaluate

18 $$\delta p(\tau ,T)\equiv \sum _{i=1}^{4}\,|p[i]|-1,$$

which is zero if, and only if, all *p*[*i*]’s are non-negative, and map it onto the $(\tau ,T)$ plane in Fig. 3. As in Fig. 2, $\delta p(\tau ,T)\ne 0$ in the light-coloured regions, and vanishes on the horizontal and diagonal lines where, as we already know from Sect. III, the classical-like probabilities can indeed be defined. We note that the negative probability test is also passed also on the vertical lines $T=n\pi$/2, $n=1,2,\ldots$ . An inspection of Eq. (17) shows that for any $\tau$, and $T=(2k+1/2)\pi$, or $T=k\pi$ there exists a suitable classical ensemble. Such ensembles, with only two paths leading to the same destination, are shown in the insets if Fig. 3. Just because these ensembles can be found in principle, does not, of course, mean that they correspond to what actually happens. To warn the reader about the misrepresentation, we crossed the insets in Fig. 3 with red lines.

Clearly, the appearance of negative “probabilities” is a sufficient, yet not necessary condition for the classical-like reasoning, based on Eq. (14), to fail. Such is the price of relying on average values, instead of the probability distributions, which contain full information about a statistical ensemble. Relying on the properties of sums of averages, rather than on the averages themselves, would be an even less precise tool, as we will demonstrate next.

## The Leggett-Garg Inequalities

Alternatively, we could use the fact that the existence of the classical-like non-negative path probabilities (11) imposes certain restrictions on the sums of the averages (14) Following^2^, one notes that in all of the four possible sequences, *Q*_3_, *Q*_2_, *Q*_1_ the sum of products $L\equiv {Q}_{1}{Q}_{2}+{Q}_{1}{Q}_{3}+{Q}_{2}{Q}_{3}$ equals 3, if all the *Q*’s have the same sign, and takes the value of −1 otherwise. It is readily seen that if the sequences occur with the probabilities in Eq. (11), also the sum of the averages (14) cannot be less than −1,

19 $$\langle L\rangle \equiv \langle {Q}_{1}{Q}_{2}\rangle +\langle {Q}_{1}{Q}_{3}\rangle +\langle {Q}_{2}{Q}_{3}\rangle \ge -1,$$

since adding the {1, 1, 1}, or $\{\,-\,1,-\,1,-\,1\}$ paths, where $\langle L\rangle =-\,1$, could only increase the sum’s value. The LGI test consists in inserting the correct quantum values (15) into (19) and looking for the values of $\tau$ and *T*, such that the inequality (19) does not hold. Thus, we will look for those values of $\tau$ and *T*, for which a sum

20 $$\delta L(\tau ,T)\equiv \langle L\rangle +1=\alpha +\beta +\gamma +1,$$

where *α*, *β* and *γ* are defined in Eq. (15), is negative. A condition $\delta L < 0$ should, therefore, signal the impossibility of assigning meaningful path probabilities *p*[*i*] in (14), in a similar way as the appearance of “negative probabilities”, discussed in the previous Section. This is, however, a less direct approach, and we ask whether it is as efficient as the tests of the previous two Sections.

**Figure 4:**
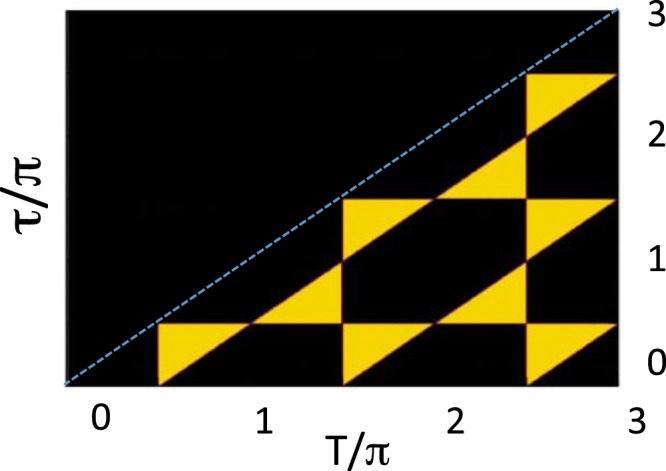
Detection of “signalling in time” by the Leggett-Garg inequality. In the light coloured areas quantum mechanical averages (15) violate the Leggett-Garg inequality (19), while in the dark coloured areas the inequality holds.

It is already known that the LGI would be satisfied on the network of the lines in Fig. 3, since on the horizontal and diagonal lines the system does demonstrate required classical-like behaviour, and on the vertical lines a suitable classical ensemble can be found, at least in principle. Accordingly, these lines divide the ($\tau$, *T*)-plane into segments, inside which the LGI is either violated (light coloured), or satisfied (black), as shown in Fig. 4. It is readily seen that the LGI test leaves much of the ($\tau$, *T*)-plane black, thus being a much less sensitive indicator of “signalling in time”, than the negative probability test of Sect. V. Such is the price of relying on the sums rules, satisfied by the averages, rather than on the averages themselves.

## Conclusions and Discussion

In summary, a sequence of quantum measurements, made on an elementary quantum system, can be described in terms of real observable paths, constructed from the virtual ones. The outcomes of previous observations can influence the results of the measurements yet to be made, provided the earlier measurements create new real scenarios, by destroying interference, which otherwise exists between the virtual paths.

A simple illustration of this principle is provided by a qubit, completing its Rabi cycle by $t=T=\pi$. In this case, two interfering paths, {3} and {4} in Fig. 1, have amplitudes of the same magnitude, but of opposite sign, ±$i\,\sin \,\tau \,\cos \,\tau$. Destructive interference prevents the system from reaching the state $|\,-\,1\rangle$. An additional measurement at $t=\tau$ makes both virtual paths real as shown if Fig. 2c, and, at $t=T$, the qubit is found in $|\,-\,1\rangle$ with a probability ${\sin }^{2}(2\tau )/4$. An earlier measurement at $t=\tau$ clearly affect the outcomes at *T*, or, if one prefers the language of ^3^, “signalling in time” occurs.

We also considered two other approaches, based on evaluation of two-time averages of the qubit’s variable. One approach assumes that the real scenarios (paths) exist at all times, and are not created by the measuring device(s). It fails, since meaningful path probabilities cannot, in general, be found where destruction of interference between virtual paths is known to take place. One exception are the vertical lines in Fig. 4, where this “negative probability test” errs by finding a spurious ensemble, consistent with the quantum mechanical averages (15), but misrepresenting the actual situation.

The second method, based on the Leggett-Garg inequalities, tests a sum rule, which the averages should satisfy in the absence of “signalling”. As suggested in^3^, the LGI provide a sufficient, but not necessary condition, and detects the quantum behaviour in far fewer cases than the negative probability test, as shown in Fig. 4. Perhaps, one reason for the popularity of the approach is the LGI’s formal similarity to the celebrated Bell’s inequality^4^. We find, however, little advantage in using the analogy, and advocate much simpler elementary methods, serving the same purpose.
